# Macrophages and autophagy: partners in crime

**DOI:** 10.1111/febs.17305

**Published:** 2024-10-22

**Authors:** Alessandra Vitaliti, Alessio Reggio, Alessandro Palma

**Affiliations:** ^1^ Department of Chemical Science and Technologies “Tor Vergata” University of Rome Italy; ^2^ Saint Camillus International University of Health Sciences Rome Italy; ^3^ Department of Biology and Biotechnologies “Charles Darwin” Sapienza University of Rome Italy

**Keywords:** autophagy, immunity, lysosome, macrophages, phagocytes

## Abstract

Macrophages and autophagy are intricately linked, both playing vital roles in maintaining homeostasis and responding to disease. Macrophages, known for their ‘eating’ function, rely on a sophisticated digestion system to process a variety of targets, from apoptotic cells to pathogens. The connection between macrophages and autophagy is established early in their development, influencing both differentiation and mature functions. Autophagy regulates essential immune functions, such as inflammation control, pathogen clearance, and antigen presentation, linking innate and adaptive immunity. Moreover, it modulates cytokine production, ensuring a balanced inflammatory response that prevents excessive tissue damage. Autophagy also plays a critical role in macrophage polarization, influencing their shift between pro‐inflammatory and anti‐inflammatory states. This review explores the role of autophagy in macrophages, emphasizing its impact across various tissues and pathological conditions, and detailing the cellular and molecular mechanisms by which autophagy shapes macrophage function.

## Graphical abstract

Autophagy is a key regulator of macrophage function, influencing critical processes like polarization, cytokine secretion, and phagocytosis. Its impact on macrophage behavior varies across different tissues and disease contexts, where it can either promote protective responses or contribute to detrimental outcomes, depending on the physiological or pathological conditions.

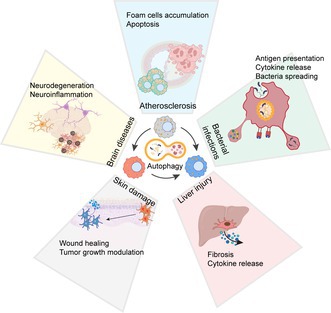

**Abbreviations**
AKI: acute kidney injury
APC: antigen‐presenting cell
ATGs: autophagy‐related proteins
ATO: arsenic trioxide
CE: cholesteryl ester
CLIP: class II‐associated invariant chain peptide
DN: diabetic nephropathy
ER: endoplasmic reticulum
GABARAPS: gamma‐aminobutyric acid receptor‐associated proteins
HCK: hematopoietic cell kinase
IFNγ: interferon gamma
LLO: listeriolysin O
Mfn2: mitofusin 2
MHC: major histocompatibility complex
MIIC: MHC‐II‐containing compartment
NAADP: nicotinic acid adenine dinucleotide phosphate
NLRP3: NLR family, pyrin domain containing 3
NOS2: inducible nitric oxide synthase
NOX/PHOX: (NADPH oxidase)‐dependent phosphorylation
PAMPs: pathogen‐associated molecular patterns
PI3KC3: class III phosphatidylinositol‐3‐kinase
PI3P: phosphatidylinositol‐3‐phosphate
PLC: bacterial phospholipases C
SYK: spleen tyrosine kinase
TFE3: transcription factor E3
TFEB: transcription factor EB
TLR2: Toll‐like receptor 2
TNF: tumor necrosis factor
ULK1/2: Unc‐51‐like kinase 1/2
VDR: vitamin D3 receptor

## Introduction

Macrophages and autophagy are intimately bound to their etiology, sharing some kind of eating function profoundly linked to their biological role in both physiology and disease. As specialized phagocytes, macrophages require a sophisticated digestion apparatus to process their diverse substrates. The macrophage diet is diverse, encompassing apoptotic cells and cellular debris in sterile immune responses, as well as pathogens in infectious settings, through a process called ‘phagocytosis’. Typically, macrophages can phagocytize pathogens and present their antigens to T lymphocytes via the major histocompatibility complex (MHC). They also clear apoptotic cells and cellular debris through a process known as ‘efferocytosis’ [1]. The apoptosis signals are recognized by a variety of receptors, which trigger intracellular signaling cascades. This leads to the engulfment of the target into a phagosome, where enzymatic digestion occurs within lysosomes [2].

Eukaryotic cells possess an intrinsic ability to initiate a specific process for digesting portions of their cytoplasm; this bulk degradative program is known as macroautophagy or simply autophagy. Through autophagy, cells can remove intracellular pathogens, damaged organelles, harmful components, and recycle macronutrients to fuel new biosynthetic pathways [3].

The capture of these materials is facilitated by double‐membraned vesicles called autophagosomes, which fuse with lysosomes to degrade their contents. Several conserved protein factors are essential for autophagy, with the most critical referred to as ‘core’ autophagy genes. Autophagy is triggered by the inhibition of mTOR, the best‐characterized negative regulator of this process. Upon mTOR inhibition, the Unc51‐like kinase 1/2 (ULK1/2) complex is recruited to specialized regions of the endoplasmic reticulum (ER) called omegasomes [4, 5, 6]. At these sites, the nucleation of the phagophore is promoted by the recruitment of components from the class III phosphatidylinositol‐3‐kinase (PI3KC3) complex I [7, 8]. This complex catalyzes the production of phosphatidylinositol‐3‐phosphate (PI3P), a lipid that attracts PI3P‐binding autophagy effectors. These effectors act as scaffolds, anchoring the ATG512/16 complex to the outer membrane of the phagophore [9, 10]. The ATG512/16 complex facilitates the conjugation of microtubule‐associated protein light chain 3 (LC3) and γ‐aminobutyric acid receptor‐associated proteins (GABARAPs) to phosphatidylethanolamine on the phagophore membrane, promoting its elongation and eventual closure [7]. The final stage of autophagy, the fusion of autophagosomes with lysosomes for cargo degradation, is coordinated by specialized proteins [11, 12].

The link between macrophages and autophagy begins early in macrophage development, significantly impacting their ability to initiate and execute their differentiation process, yet also impacting mature macrophage behavior in a multitude of contexts. After exiting the bone marrow, monocytes typically differentiate into macrophages upon entering tissues. In the absence of differentiation signals, monocytes undergo programmed cell death. However, the presence of differentiation signals initiates a process involving the inhibition of caspase‐3 operated by the autophagy machinery. This has been demonstrated through experiments using knockdown of autophagy master regulators and autophagy inhibitors [13]. Indeed, under these conditions, circulating monocytes underwent apoptosis even in the presence of macrophage differentiation signals.

In macrophages, autophagy intersects with several critical immune functions. One of its primary roles in these phagocytes is to regulate inflammation and pathogen clearance. Through a process known as xenophagy, macrophages use the autophagy machinery to degrade intracellular pathogens, thereby playing a direct role in host defense [3]. Additionally, autophagy is involved in antigen presentation, aiding in the processing and presentation of antigens to T cells, which links innate and adaptive immunity [14].

Autophagy also modulates cytokine production, which is essential for controlling the inflammatory response [15, 16]. For instance, autophagy‐related proteins (ATGs) can influence the production of key cytokines such as IL1β and TNFα [15, 17, 18]. By regulating the degradation of inflammasome components and reactive oxygen species (ROS), autophagy helps maintaining a balance between pro‐inflammatory and anti‐inflammatory responses, which is crucial for preventing excessive tissue damage during infection or injury [19, 20]. Within this context, the role of autophagy in macrophage differentiation and polarization is noteworthy. Macrophages can polarize into pro‐inflammatory (M1) or anti‐inflammatory (M2) phenotypes, depending on the signals they receive and the surrounding environment [21, 22]. Autophagy influences the polarization process by regulating key signaling pathways and metabolic processes.

In this review, we will discuss the role of autophagy in macrophages, with a special focus on macrophage autophagy across different tissues and organs and in distinct pathological conditions. We will highlight the cellular and molecular mechanisms by which autophagy determines macrophage functioning, and how the alteration of the autophagic machinery can influence macrophage behavior.

## Macrophage autophagy in antigen presentation

The complex interplay between macrophage activation and autophagy plays a crucial role in the immune response to bacterial infections and the maintenance of cellular homeostasis. As a form of specialized phagocytes, macrophages are responsible for both infective and sterile immune responses. When functioning as antigen‐presenting cells (APCs), macrophages predominantly present antigens on MHC class II (MH‐CII) molecules. This process can function through distinct processing pathways, including autophagy [23, 24, 25, 26]. Within this context, a distinct process named LC3‐associated phagocytosis, play a fundamental role, being responsible of the microbe digestion, and also stabilizing and maintaining antigens for longer periods, thus prolonging MHC‐II presentation [27].

The exact process by which intracellular and extracellular pathogens are delivered to the autophagic machinery for their degradation is still under investigation, but some hypotheses point at mechanisms that are similar to those characterizing selective autophagy of endogenous cargoes [28].

**Fig. 1:**
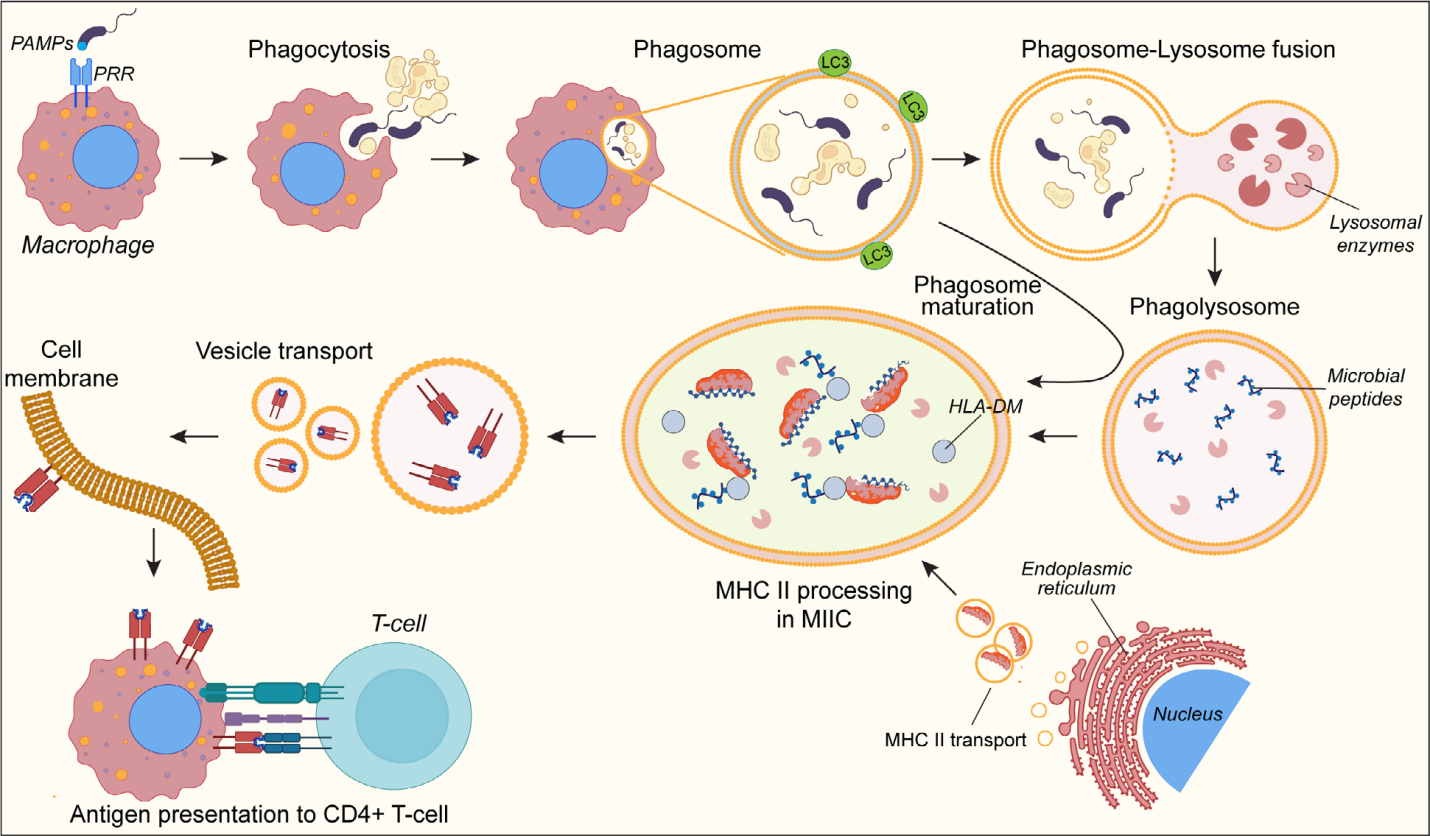
Mechanism of macrophage phagocytosis for the antigen presentation. Macrophages recognize pathogen‐associated molecular patterns (PAMPs) on microbes through pattern recognition receptors (PRRs) and phagocytize them. Phagocytized material is engulfed inside a phagosome, which fuses with lysosomes for the cargo digestion by lysosomal enzymes. This process helps in forming small peptides to be loaded onto MHC class II molecules for antigen presentation to T cells. Precursors of MHC‐II molecules from the endoplasmic reticulum are transported via vesicles to the endosomal compartment known as MHC class II‐containing compartment (MIIC), where they are cleaved and bind the microbial peptides. The complex between MHC‐II and peptides is transported to the cell membrane through vesicles, where it is exposed for antigen presentation.

One possible process that links the autophagy of extracellular pathogens to MHC class II‐mediated antigen presentation is provided in Fig. 1. In this view, macrophages recognize bacteria through pattern recognition receptors (PRRs) such as toll‐like receptors, that have been shown to induce the formation of phagolysosomes [29], and scavenger receptors. These receptors bind to pathogen‐associated molecular patterns (PAMPs) on the bacterial surface [30]. Once attached, the macrophage engulfs the bacteria by extending its plasma membrane around the bacterium, thus forming a phagosome. The phagosome can then fuse with lysosomes, for the degradation of the internalized cargo, to produce small peptides that can be loaded onto the MHC‐II molecule [31].

Class II molecules are synthesized in the ER and associate with the invariant chain (Ii), which prevents premature binding of peptides. In the endosomal/lysosomal compartments, called MHC‐II‐containing compartment (MIIC) in APCs, the invariant chain is cleaved, leaving a small fragment called CLIP (class II‐associated invariant chain peptide) in the peptide‐binding groove. Within the MIIC, HLA‐DM facilitates the exchange of CLIP for the peptides, allowing them to bind to the MHC‐class II molecules. The peptide–MHC‐class II complex is then transported to the cell surface via vesicles. On the cell surface, the peptide–MHC class II complex is presented to CD4+ T helper cells [32]. This interaction is crucial for the activation of T cells, which then coordinate the immune response by secreting cytokines, helping B cells producing antibodies, and recruiting other immune cells.

It has been demonstrated that peptides loaded onto class II molecules not only derive from internalized external antigens. Indeed, a 20% of these peptides derive from intracellular material [33], which could involve the functioning of autophagy in the mechanism of peptide–MHC‐II complex formation [31]. It is also essential to keep in mind that this is a simplified representation of the cellular mechanism by which autophagy intersect MHC‐II antigen presentation. In reality, each antigen‐presenting cell contains multiple early endosomes and antigen‐processing compartments. These compartments form a spectrum, with varying levels of the key components needed to produce peptide–MHC‐II complexes within each cell. Hence, the specific antigen‐processing compartments utilized by different antigens can differ significantly.

## Macrophage autophagy in bacterial infections

**Fig. 2:**
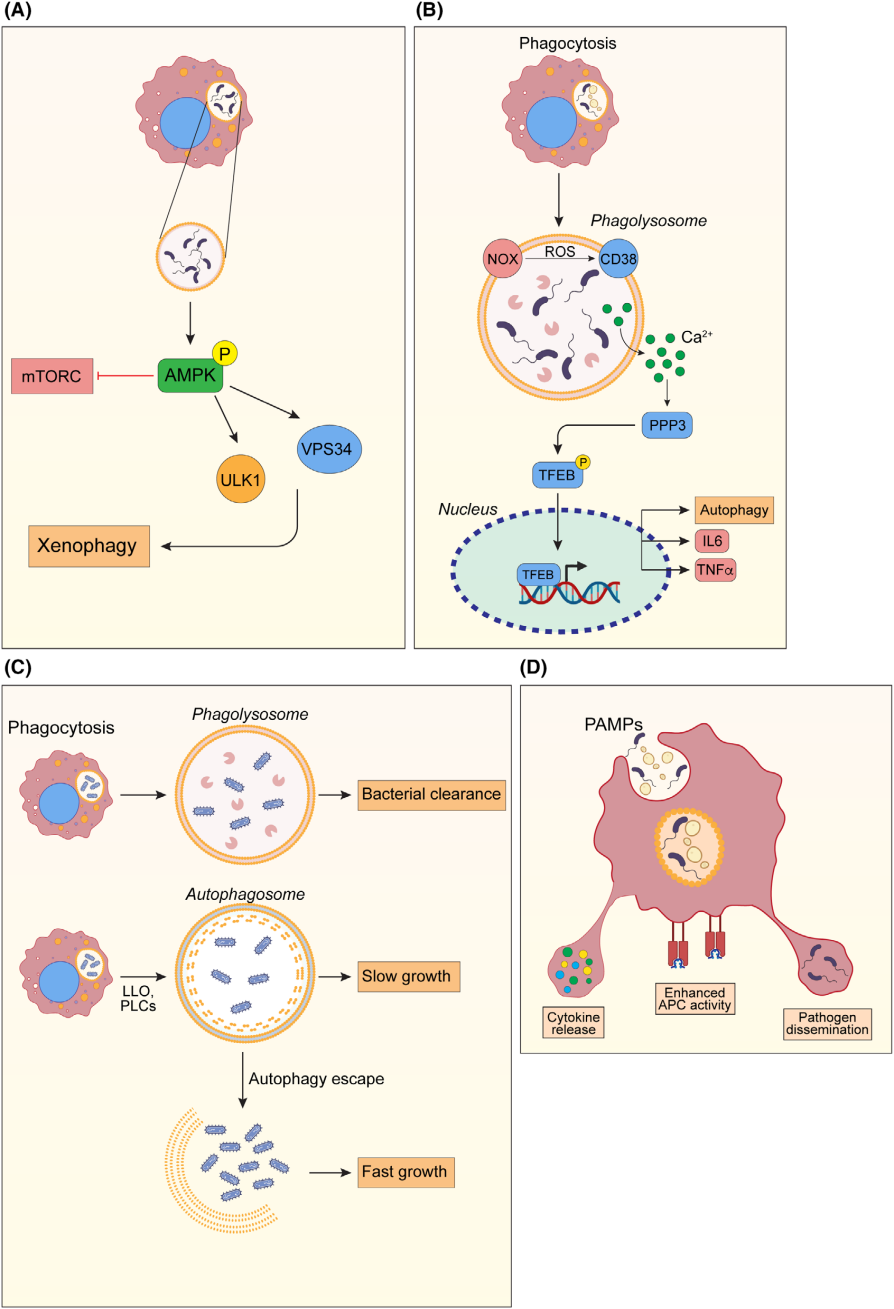
Autophagy response to bacterial infections in macrophages. (A) Bacteria inside vesicles can activate AMPK, which in turn inhibits mTOR and activates ULK1 and VPS34 to activate xenophagy. (B) After phagocytosis, bacteria are engulfed into phagolysosomes, where the release of calcium activates TFEB, which translocate into the nucleus. This leads to enhanced autophagy and the production of inflammatory cytokines. (C) Cellular mechanism by which *Listeria monocytogenes* can damage autophagosomes and activate a persistent infection. (D) Mechanism of eructophagy with the release of cytokines, pathogens, and increased antigen presentation function by macrophages upon bacterial infection.

Autophagy represents one of the key initial steps characterizing macrophage response to bacterial infections. In such specific form autophagy is referred to as xenophagy and one of the initial steps of this process is the phosphorylation of AMPK, a well‐known activator of autophagy. It has been demonstrated that bacteria detection triggers AMPK activity, which in turn leads to the release of mTORC1‐mediated repression of the autophagy pathway that is activated by a mechanism different from the bulk autophagy [34] (Fig. 2A). Another notable example is represented by TFEB (transcription factor EB) and TFE3 (transcription factor E3), two master genes for lysosome biogenesis [35], that have been demonstrated important in macrophage activation during bacterial phagocytosis [36]. Macrophages deficient in TFEB and TFE3 fail to adopt a pro‐inflammatory phenotype in response to bacterial infection. In physiological conditions, macrophage activation upon bacterial infections involves the NOX/PHOX (NADPH oxidase)‐dependent oxidative burst, which facilitates the nuclear translocation of TFEB through a ROS (reactive oxygen species)‐dependent mechanism, further influenced by CD38 and NAADP (nicotinic acid adenine dinucleotide phosphate) (Fig. 2B). The chelation of intracellular calcium and inhibition of PPP3/calcineurin prevent TFEB activation, highlighting the role of calcium signaling in this process. Consequently, TFEB and TFE3 activation are critical for the induction of pro‐inflammatory cytokines like IL6 and TNFα, underpinning the importance of these factors in macrophage‐mediated inflammation and various disease contexts, including atherosclerosis and obesity.

Reactive oxygen species production play a fundamental role in the regulation of autophagy in macrophages, also influencing mitochondrial dynamics. Within this context, the protein mitofusin 2 (Mfn2) has been shown as an important factor in macrophage mitochondrial dynamics [37]. Mfn2 is essential for adapting mitochondrial respiration to stress conditions and for ROS production during pro‐inflammatory activation. Mfn2 deficiency impairs the production of pro‐inflammatory cytokines, nitric oxide, and disrupts processes like autophagy, apoptosis, phagocytosis, and antigen processing. This deficiency leads to an inability to effectively combat infections from pathogens like *Listeria monocytogenes* and *Mycobacterium tuberculosis*, highlighting the critical role of Mfn2 in macrophage‐mediated immune responses, and the crosstalk between autophagy and the response to pathogens.

In the study of macrophage autophagy in response to bacterial infections, some pathogens provide excellent models. Some bacteria are indeed capable of adopting immune evasion behaviors, adapting to the host mechanisms of defense. One such example is *L. monocytogenes*, that uses listeriolysin O (LLO) to escape from the phagosome, yet it faces autophagic targeting in LLO‐damaged phagosomes [38] (Fig. 2C). Interestingly, *L. monocytogenes* can evade autophagy through mechanisms such as actin‐based motility and bacterial phospholipases C (PLCs). Moreover, a subset of bacteria can form the so‐called ‘Spacious *Listeria*‐containing Phagosomes’ (SLAPs), which do not mature and permit slow bacterial growth, leading to a persistent infection.

In bacterial infections, macrophages are also capable to respond by releasing material in the extracellular space, a biological event named eructophagy [39]. In this process, partially digested pathogen‐associated molecular patterns (PAMPs) are released extracellularly from the mature phagolysosome that fuses to the cell membrane, leading to an amplification of the local inflammation [40]. Eructophagy is induced by pro‐inflammatory stimuli and is negatively regulated by IL4 and mTOR (Fig. 2D). In fact, eructophagy depends on key autophagy proteins, suggesting a complex role for autophagy in modulating inflammation beyond pathogen degradation.

Macrophage‐mediated autophagy has also a pivotal role in counteracting viral infections, especially for those viruses that use the endoplasmic reticulum (ER) as a niche for their replication and assembly. In such selective degradation event, autophagy digests portion of ER containing viral foci and it can be referred to as ER‐phagy [3, 41, 42]. Macrophage selective ER‐phagy alleviates ER stress, eliminates viral colonized portion of ER, and supports innate immune response upon viral infection in multiple tissues, including liver, respiratory tract, and brain [43].

Altogether, the studies on macrophage response to bacterial infection through the activation of the autophagy machinery underscore an intricate portrait in which the activation and functional adaptation of macrophages are profoundly linked to autophagy dynamics.

## Macrophage autophagy in atherosclerosis

Recent research has revealed the critical roles of autophagy and macrophages in the progression and potential treatment of atherosclerosis, a leading cause of cardiovascular disease worldwide. Various studies have explored different compounds and mechanisms that influence autophagy and macrophage behavior, revealing significant insights into their contributions to atherogenesis and plaque stability. In both physiology and atherosclerosis states, macrophages can be found as foam cells, which are cells containing cholesterol and low‐density lipoproteins, and that show an M2‐like polarized phenotype [44]. Despite the clearance and metabolism of cholesterol has been for a long attributed to the action of neutral CE hydrolases, recent advances have clarified the contribution of autophagy in the hydrolysis of lipid droplets in cholesterol‐loaded macrophages [45].

**Fig. 3:**
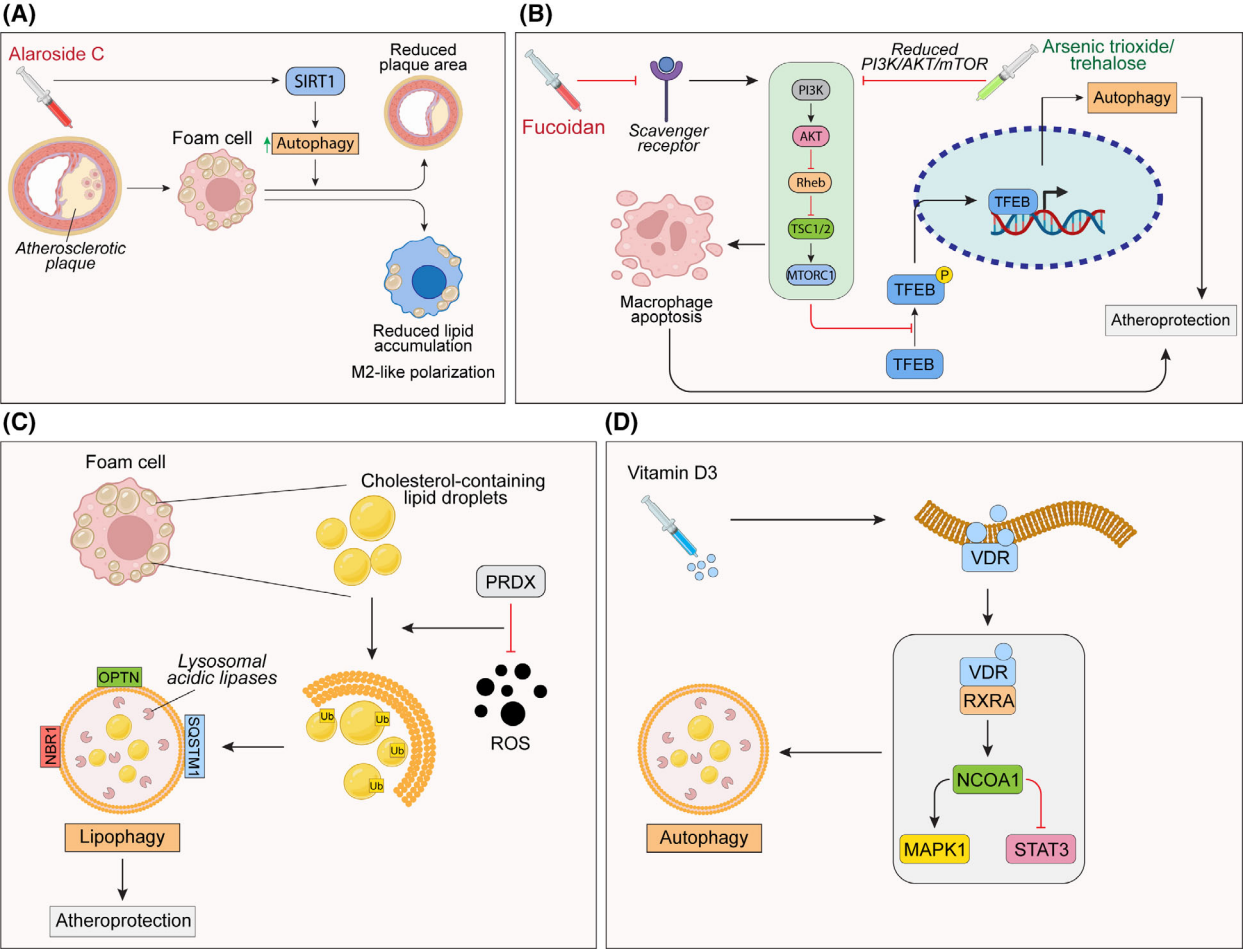
Macrophage autophagy in atherosclerosis. (A) Influence of the SIRT1 pathway upon drug treatment on the regulation of atherosclerotic plaques from foam cells. (B) Effect of mTOR pathway and TFEB activation on autophagy in the regulation of macrophage response within atherosclerosis settings. (C) Regulation of the selective autophagy of lipid droplets (lipophagy) by oxidative stress in atherosclerosis. (D) Molecular effects of vitamin D3 administration on foam cells autophagy regulation.

One recent study focused on Araloside C, a natural compound with anti‐inflammatory properties, that has been object of investigation on macrophage polarization and autophagy in both *in vivo* and *in vitro* models of atherosclerosis [46]. The authors found that this compound significantly reduced plaque area and lipid accumulation in macrophages exposed to oxidized low‐density lipoprotein, which was accompanied by a switch to the M2 macrophage phenotype and an increase in autophagosome formation. Importantly, the protective effects of Araloside C were linked to the activation of the Sirt1‐mediated autophagy pathway (Fig. 3A). In fact, the inhibition of autophagy or Sirt1 significantly diminished the benefits from the pharmacological treatment, highlighting the importance of this pathway in mitigating foam cell formation and atherosclerosis.

In another study, it has been found that arsenic trioxide (ATO) activated the nuclear TFEB, which enhanced autophagy and lysosomal biogenesis [47]. This activation was linked to a reduction in the PI3K/AKT/mTOR signaling pathway, a key regulator of autophagy. The study also found that ROS played a crucial role in ATO's effects, as antioxidants could block these actions (Fig. 3B). Analogous effects were obtained using trehalose, an autophagy‐inducing disaccharide able to increase TFEB activity [48], which led to an increased autophagy and a subsequent atheroprotection.

As the mTOR activation constitute a key player in autophagy regulation, it is not surprisingly that many studies rely on the targeting of mTOR and its downstream and upstream effectors, including scavenger receptors. Indeed, due to the main role of macrophages as phagocytizing cells, their scavenger receptors have been extensively studied for their playing crucial roles in recognizing and clearing modified lipoproteins, pathogens, and cellular debris, thereby contributing to immune defense and maintaining tissue homeostasis. Within this context, a study investigated the use of fucoidan, a class A scavenger receptor agonist, under conditions of endoplasmic reticulum stress [49]. The study found that this compound inhibited autophagy by activating the mTOR pathway, which led to increased apoptosis in macrophages (Fig. 3B).

Besides noncanonical autophagy mechanisms, macrophages can also rely on selective autophagy. This is a form of autophagy in which distinct cargoes can activate a dedicated degradation/recycling pathway, often ubiquitin‐dependent, through the use of specific receptors [50]. In foam cells, lipophagy, a selective autophagy targeting lipid droplets, stands out and has been object of recent studies in atherosclerosis settings. For instance, a recent report identified a list of proteins associated with lipid droplets in macrophage foam cells and demonstrated that knocking down specific genes impaired cholesterol efflux [51]. Their results link the enhancement of lipophagy to promote cholesterol removal and reduce atherosclerotic burden, which could have possible therapeutic implications in atherosclerosis. Indeed, the enhancement of autophagy has been promoted as a potential therapeutic target in atherosclerosis settings, using nanoparticles that increase the acidification of lysosomes, hence improving their ability to clear lipid droplets aggregates [52]. Moreover, autophagy activation through the lipophagic flux was found to be crucial in oxidative stress condition, a significant factor in atherosclerosis [53]. Research demonstrated that the antioxidant enzyme PRDX1 is vital for this process, as its deficiency led to an impaired autophagic flux, and disrupted cholesterol homeostasis in macrophages (Fig. 3C).

Additionally, macrophage autophagy plays a fundamental role in adaptive immunity within atherosclerotic plaques. In fact, macrophages in these plaques act as APCs that regulate adaptive immune responses. An important entity, the spleen tyrosine kinase (SYK), regulates autophagy and MHC class II expression in response to oxidized LDL [54]. This regulation impacts the presentation of antigens to T cells, influencing adaptive immune responses and potentially contributing to the atherogenesis.

Finally, the targeting of foam cells, which results augmented in atherosclerosis, has been object of investigation using exogenous administration of vitamin D3, a compound that is known to inhibit foam cell formation [55]. The activation of the vitamin D3 receptor (VDR) pathway was able to achieve such goal by activating the autophagic flux via the expression of MAPK1 and the inhibition of STAT3 (Fig. 3D).

## Macrophage autophagy in healthy and diseased liver

**Fig. 4:**
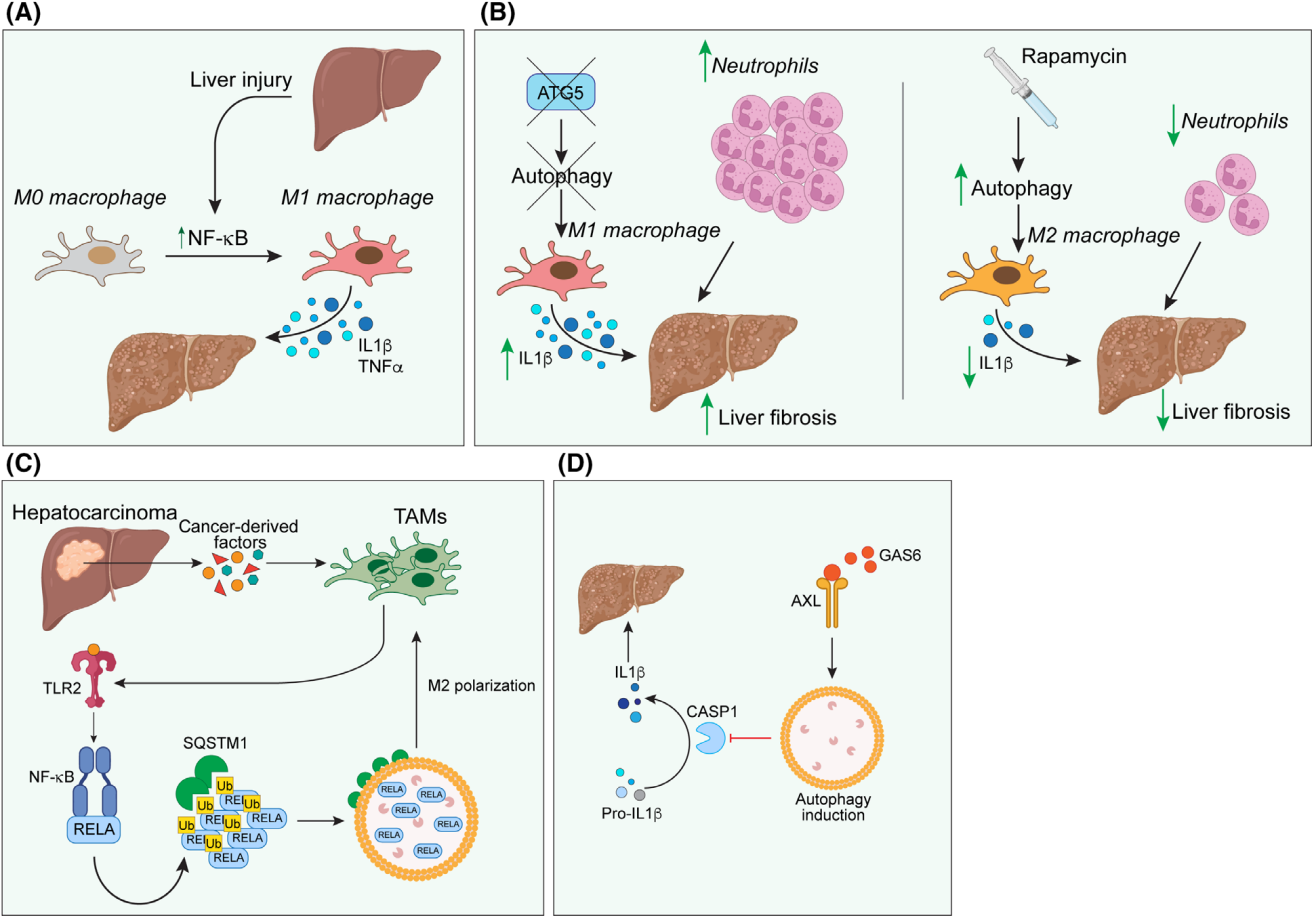
Macrophage autophagy in the liver. (A) Cellular mechanism by which fibrosis induces M1 macrophage polarization, leading to the release of pro‐inflammatory cytokines, such as IL‐1β. (B) Impaired autophagy results in enhanced M1 macrophage polarization, characterized by the release of inflammatory cytokines, increased neutrophil recruitment, and exacerbation of liver fibrosis. In contrast, upregulation of autophagy promotes M2 macrophage polarization, leading to reduced inflammatory cytokine levels, fewer neutrophils, and attenuation of liver fibrosis. (C) In hepatocarcinoma, cancer‐related factors activate tumor‐associated macrophages (TAMs) via upregulation of the TLR pathway, which enhances autophagy and drives TAMs towards an M2‐like phenotype. (D) Autophagy activation via the GAS6‐AXL axis inhibits caspase‐mediated cleavage of pro‐IL‐1β into its active form, thereby reducing liver fibrosis.

Liver‐resident macrophages are known as Kupffer cells and are responsible for maintaining the tissue homeostasis. Like many other tissue‐resident macrophages, they originate from the yolk sac and colonize liver during embryogenesis. However, in certain circumstances, including acute and chronic injuries, circulating monocyte can be recruited and differentiate into macrophages to play different functions [56]. Indeed, in chronic disease states of the liver, macrophages can exert a dual role, including the perpetuation of the inflammatory state by a continuous activation of stellate hepatic cells, and the resolution of inflammation through the degradation of extracellular matrix and the release of anti‐inflammatory mediators [57]. Typically, upon injury liver macrophages polarize towards an M1 state through the activation of NF‐κB, secreting pro‐inflammatory cytokines such as IL1β and TNFα [58], thus promoting fibrosis. It has been reported in mouse models of liver fibrosis that autophagy can have protective effects by limiting the production of pro‐inflammatory cytokines [59]. The knock out of the autophagy master gene Atg5 increases the production of IL1 cytokine family members and favors the recruitment of neutrophils to the site of injury. By contrast, the forced induction of autophagy by rapamycin treatment decreases the levels of IL1 and the recruitment of other inflammatory immune cells, thus limiting the liver fibrosis (Fig. 4A,B). The evidence that pro‐inflammatory macrophages exacerbate liver injury due to the repression of autophagy comes from another report showing that aged mice displayed an increased fibrosis accompanied by a shift in M1 macrophages together with an impaired autophagy triggered by ATG5 repression [60]. Restoration of ATG5 levels in acute liver injury mice was able to rescue the M2 macrophage polarization and helped to reduce the number of apoptotic and necrotic hepatocytes.

The activation of NF‐κB pathway is vital also in liver cancer settings, where TAMs are known to regulate the tumor inception and growth [61]. In these settings, in response to hepatoma‐derived factors, macrophages use the toll‐like receptor 2 (TLR2) pathway to get the NF‐κB/RELA cytosolic ubiquitination, which is then degraded through a SQSTM1/p62‐dependent mechanism, thus favoring an M2‐like polarization (Fig. 4C).

In another mouse model of toxin‐induced liver injury from D‐galactosamine/ lipopolysaccharide, the authors showed that the lack of autophagy (ATG5 KO) in liver macrophages led to increased injury due to the caspase 1‐dependent cleavage of pro‐IL1β to its active form [62]. This cleavage can be prevented by the activation of AXL, a receptor tyrosine kinase that induces autophagy in macrophages. After the interaction with its ligand GAS6, the subsequent inhibition of NLRP3 (NLR family, pyrin domain containing 3) inflammasome activation prevents IL1β maturation, hence improving the hepatic inflammatory response (Fig. 4D).

Other studies on the broader implications of macrophage autophagy have shown their crucial role in regulating inflammation. For instance, impaired autophagy in macrophages was linked to increased hepatic inflammation and liver injury in the context of obesity and a high‐fat diet [63]. This impairment led to altered macrophage polarization, with an increase in pro‐inflammatory M1 and a decrease in anti‐inflammatory M2 phenotypes.

## Macrophage autophagy in healthy and diseased skin

**Fig. 5:**
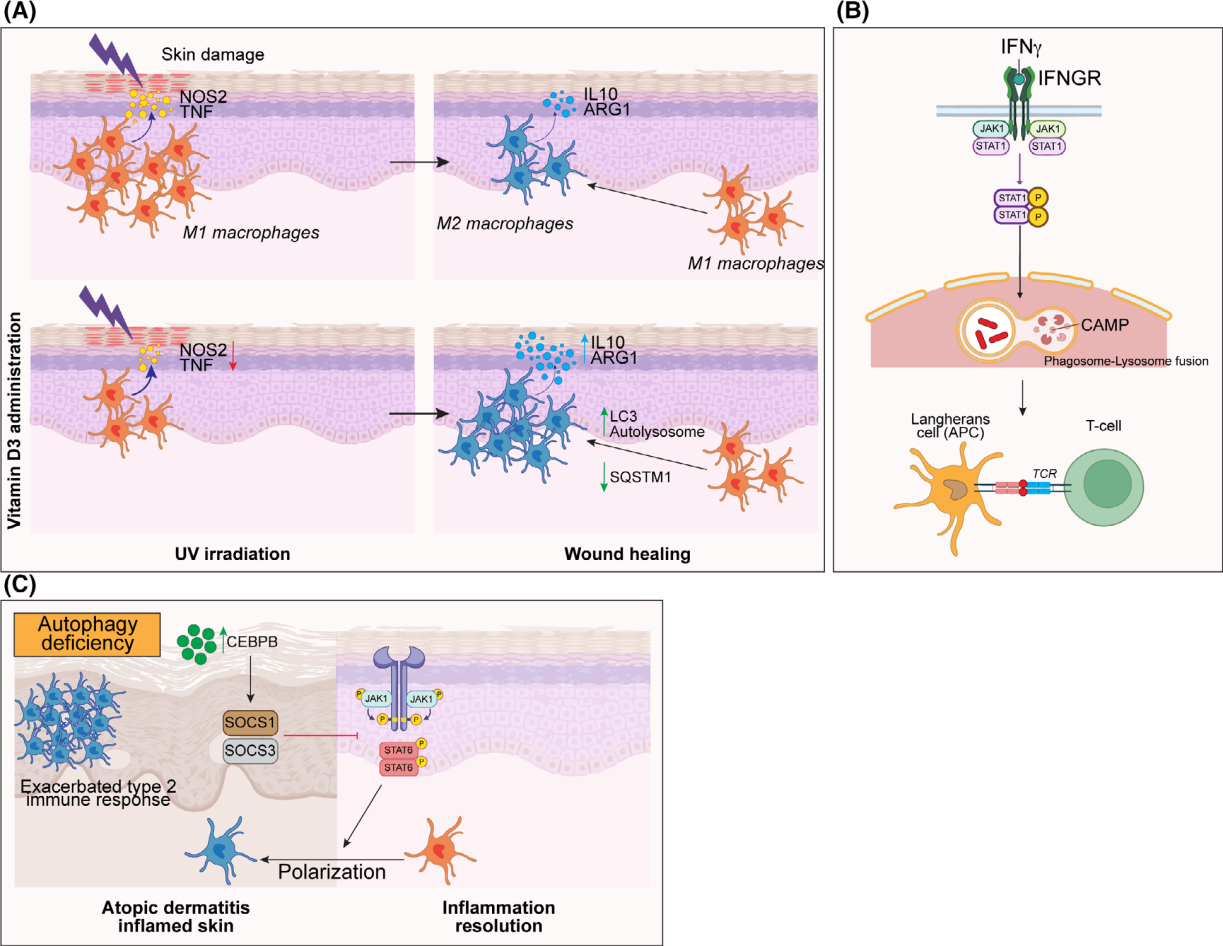
Cellular and molecular mechanisms of macrophage autophagy in skin. (A) In skin damage induced by UV irradiation, macrophages polarize towards an M1‐like phenotype, secreting inflammatory cytokines. Vitamin D3 administration reduces the population of pro‐inflammatory macrophages. During wound healing, macrophages transition to an M2‐like phenotype, and vitamin D3 promotes this switch, increasing autolysosome formation. (B) In the skin, IFNγ enhances the antigen‐presenting activity of Langerhans cells, which in turn activates the autophagy machinery. (C) In atopic dermatitis, decreased autophagy limits the exacerbated type 2 immune response, facilitating the resolution of inflammation.

Skin‐resident macrophages, known as Langerhans cells, are a specialized class of myeloid cells sharing features with dendritic cells, that continuously recognize and sequester external antigens and reorganize epidermal layering of keratinocytes [64]. They also establish a crosstalk with other immune cells to maintain tissue homeostasis and regulate cutaneous immunity [65]. In the skin, macrophages are responsible for the immune response to several types of insults, including UV irradiation, and for the dermatological chronic disease. Indeed, upon inflammation induced by sunburn, macrophages polarize towards an M2 state, secreting IL‐10 to dampen inflammation [66] (Fig. 5A). It has been reported that the administration of cholecalciferol D3 mitigates skin inflammation by reducing the expression of inducible nitric oxide synthase (NOS2) and tumor necrosis factor (TNF), which is associated to a switch towards arginase (ARG1) metabolism in macrophages. This process is promoted by an enhanced autophagy, with increased expression of LC3 and degradation of p62, coupled with the autolysosome formation [67].

In epidermal immune responses against *Mycobacterium leprae*, Langerhans cells act as antigen‐presenting cells, interacting with T lymphocytes [68]. This is achieved by the induction of autophagy via the interferon gamma (IFNγ) pathway, which is followed by the fusion of the pathogen‐containing phagosome with lysosomes, and the intra‐lumen release of cathelicidin, a well‐known antimicrobial peptide (Fig. 5B).

In other epidermal contexts, macrophage autophagy could play a detrimental role. In a model of atopic dermatitis, autophagy deficiency leads to a decreased skin inflammation, limiting the exacerbated type 2 immune response observed in this pathology. Indeed, the accumulation of CEBPB is able to inhibit M2 macrophage polarization through the upregulation of SOCS1/3 which in turn inhibits JAK1/STAT6 pathway [69] (Fig. 5C).

## Macrophages and autophagy in healthy and diseased kidney

Macrophage autophagy is also crucial in maintaining kidney health, particularly in the context of diabetic nephropathy (DN), a common and severe complication of diabetes. The infiltration of macrophages into kidney tissue is a key pathological feature of DN, and recent studies have shed light on the intricate relationship between macrophage autophagy and their adhesion and migration responses. In a study exploring this relationship, researchers observed that diabetic conditions significantly impair autophagy in macrophages, which in turn enhance their adhesion and migration capabilities [70]. In diabetic rats, increased renal injury was correlated with a suppression of autophagy, paralleled by an increased presence of CD68‐positive macrophages in the kidney tissue, indicating enhanced macrophage infiltration.

Another study investigated the role of hematopoietic cell kinase (HCK) in promoting kidney fibrosis in chronic kidney disease (CKD) [71]. The research showed that HCK is highly expressed in pro‐inflammatory macrophages in diseased kidneys. By using HCK‐knockout models and a HCK inhibitor, the study demonstrated that reducing HCK activity decreases macrophage pro‐inflammatory polarization, proliferation, and migration, and that this effect was linked to HCK's interaction with autophagy‐related proteins, which inhibits autophagy in macrophages.

In acute kidney injury (AKI), macrophage autophagy is important in regulating renal fibrosis, contributing to the inflammation‐related condition. It has been recently demonstrated that the depletion of autophagy in kidney macrophages exacerbate the inflammation via an impaired crosstalk with tubular epithelial cells [72]. This was further confirmed by a recent study in which the lack of macrophage autophagy was linked to a more severe AKI phenotype, with abnormally high levels of serum creatinine, and elevated expression of inflammatory factors [73].

## Macrophages and autophagy in brain and neurodegeneration

Brain‐resident macrophages are known as microglia and exert important functions in tissue homeostasis [74]. They are capable of clearing cellular debris and establish a crosstalk with other brain cells, including neurons and oligodendrocytes, to ensure the correct tissue functioning. Unlike circulating macrophages, microglia are not able to polarize in the typical M1/M2 phenotypes [75]. They have been rather classified into other type of functional categories, including homeostatic and adaptive microglia, based on their transcriptional profile, morphology, and function [76, 77]. However, circulating macrophages, often reported as perivascular macrophages, can reach the brain tissue in certain conditions, including brain injury and neurodegeneration, exerting immune response functions [78].

**Fig. 6:**
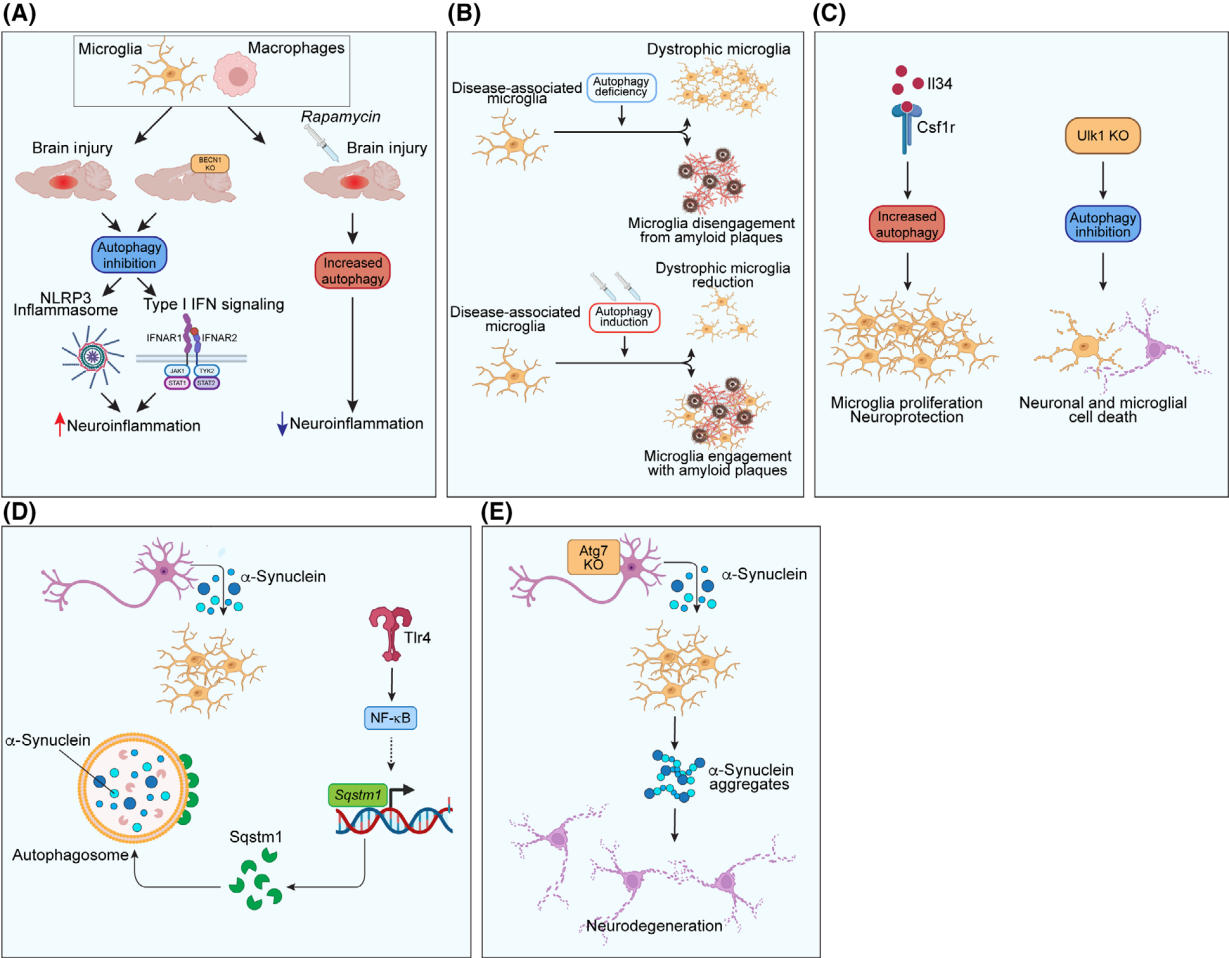
Cellular and molecular mechanisms of macrophage autophagy in the brain. (A) In the injured brain, autophagy inhibition activates the inflammasome response, leading to increased neuroinflammation, whereas autophagy induction reduces neuroinflammation. (B) In diseased microglia, autophagy activation promotes the clearance of β‐amyloid plaques. (C) Autophagy in microglia, through the activation of CSF1R pathways, triggers neuroprotective effects in neurons. (D) The TLR4/NF‐κB pathway upregulates SQSTM1, facilitating the clearance of α‐synuclein. (E) In the absence of autophagy, α‐synuclein aggregates form, promoting neurodegeneration.

The role of autophagy in microglial cells and macrophage is still under investigation, and many mechanisms remain unclear. However, several reports highlighted the effect of autophagy in these cells, and in particular, its role in modulating the response to injury and inflammation. A recent study described how autophagy can profoundly affect the ability of these cells to modulate inflammation after acute brain injury in mouse models [79]. In particular, the authors showed that upon injury, and in autophagy deficiency conditions given by the knockout of Beclin‐1 (BECN1), both macrophages and microglia increased their levels of NLRP3 inflammasome and type I IFN responses, which resulted in the perpetuation of inflammation in the site of injury. Conversely, when they pharmacologically induced autophagy in brain‐injured mice, microglia and macrophages were able to dampen inflammation, with decreased levels of NLRP3 (Fig. 6A). Restoration on the proper autophagy levels has been also found beneficial in neurodegenerative context, such as Alzheimer's disease, where autophagy has been linked to positive effects on the removal of amyloid plaques [80]. The inhibition of autophagy in brain‐resident macrophages promotes a senescent phenotype of microglia, their disengagement from amyloid plaques, with aggravation of the disease phenotype in a mouse model (Fig. 6B). Conversely, pharmacological restoration of autophagy with senolytic drugs helps in removing senescent and dysfunctional microglia and ameliorates the pathology. In general, the impairment of autophagy can lead to the inability of microglia to clear amyloid plaques, the accumulation of protein aggregates that are not cleared by glial cells, and the subsequent death of neurons [81].

Microglia functioning was also found altered in aging and other neurodegenerative conditions. In particular, aged microglia are characterized by an increased canonical autophagy, following an impaired functioning in ERK1/2, Akt, and AMPK, all downstream signals of CSF1R [82]. Such activation exerts neuroprotective effects, while upon the autophagy removal by Ulk1 knock out, microglia population resulted decreased (Fig. 6C). This particular class of microglia is dependent on IL34, which points at autophagy as a central hub in neurodegenerative disorders, using IL34 as modulator of microglia functioning.

Autophagy is also involved in the removal of cellular debris and protein aggregates in physiological and pathological conditions. One example is the clearance of alpha‐synuclein aggregates produced by the release of this soluble protein by neurons. In Parkinson's disease, alpha‐synuclein form aggregates the are cleared by microglia (Fig. 6D,E). Upon the release of this protein, microglia are recruited, and engulf alpha‐synuclein in phagosome, activating the TLR4 receptor, which acts via NF‐κB pathway to increase the expression of SQSTM1/p62, and initiate a p62‐driven autophagic pathway [83]. By contrast, in mouse models expressing human alpha‐synuclein, the disruption of the autophagy machinery was seen to cause midbrain dopaminergic neuron degeneration.

## Conclusion

This review underscores the pivotal role of autophagy in shaping macrophage function across various tissues and disease states. From bacterial infections to chronic conditions like atherosclerosis, liver fibrosis, and neurodegenerative diseases, autophagy emerges not just as a regulator of macrophage behavior, but as a key orchestrator of both immune responses and tissue repair processes.

In the context of bacterial infections, macrophage‐mediated xenophagy is essential for pathogen clearance, while also contributing to antigen presentation and fine‐tuning inflammatory responses. The potential therapeutic implications of this are significant, as impaired macrophage autophagy could lead to increased susceptibility to infections. As such, enhancing this process may offer new strategies to combat infectious diseases. By promoting the clearance of intracellular pathogens and modulating inflammation, boosting macrophage autophagy could limit bacterial survival while preventing tissue damage caused by excessive inflammation. This dual functionality presents a promising avenue for therapeutic exploration.

As central hubs in immune responses, macrophages are not only involved in infection control but also play a critical role in sterile inflammation. Autophagy has been shown to regulate macrophage polarization through distinct molecular pathways, influencing their transition between pro‐inflammatory and anti‐inflammatory states. This ability to guide macrophage behavior places autophagy at the heart of both acute and chronic inflammation resolution. For instance, in atherosclerosis, autophagy's influence on macrophage polarization and foam cell formation suggests that targeting autophagic pathways could slow or even reverse disease progression. This leads us to speculate that autophagy may serve as a broader therapeutic target, with potential applications across a range of inflammatory and degenerative conditions.

However, while the therapeutic potential of autophagy modulation in macrophages is exciting, the complexity of its effects warrants caution. Autophagy can have opposing outcomes depending on the tissue or disease context. For instance, in liver fibrosis or skin diseases, autophagy might either promote healing or exacerbate tissue damage, depending on the timing and extent of its activation, and on the distinct induced intracellular signaling cascades. This highlights the need for precision in targeting autophagy‐related pathways, as simply enhancing or suppressing autophagy may not be enough. Instead, interventions will likely need to be finely tuned to the specific disease environment to avoid unintended consequences.

Autophagy dysfunction in brain‐resident macrophages also plays a crucial role in neurodegenerative diseases such as Alzheimer's and Parkinson's. Microglial autophagy is essential for maintaining neuronal homeostasis and modulating neuroinflammation. In Alzheimer's disease, for example, microglial‐mediated autophagy significantly contributes to the clearance of amyloid plaques and protein aggregates, underscoring the complex interplay between autophagy and neuroinflammatory responses. Targeting autophagy components in microglia could hold promise for mitigating neurodegeneration, though future studies must ensure that such interventions are safe and effective in clinical settings.

In light of this, future studies should focus on understanding the context‐specific roles of macrophage autophagy across different tissues and disease states. While there is immense therapeutic potential, it will be critical to deepen our understanding of how autophagy operates in distinct macrophage subtypes and pathological environments. A more nuanced approach to modulating autophagy could unlock new possibilities in treating inflammatory and degenerative diseases, but such strategies must be approached with careful consideration to avoid detrimental effects. Future research should continue to explore these pathways to better elucidate how autophagy influences macrophage function and to translate these findings into effective clinical interventions.

## Conflict of interest

The authors declare no conflict of interest.

## Author contributions

AP, AR, and AV contributed to conceptualization, visualization, formal analysis, and resources, writing—original draft, writing—review and editing. AP contributed to supervision and project administration.
